# Intensity of perinatal care for extremely preterm babies and outcomes at a higher gestational age: evidence from the EPIPAGE-2 cohort study

**DOI:** 10.1186/s12887-019-1856-1

**Published:** 2020-01-07

**Authors:** Andrei Scott Morgan, Babak Khoshnood, Caroline Diguisto, Laurence Foix L’Helias, Laetitia Marchand-Martin, Monique Kaminski, Jennifer Zeitlin, Gérard Bréart, François Goffinet, Pierre-Yves Ancel

**Affiliations:** 1 Université de Paris, Epidemiology and Statistics Research Center/CRESS, INSERM (U1153 – Obstetrical, Perinatal and Pediatric Epidemiology Research Team (EPOPé)), INRA, Hôpital Tenon, Bâtiment Recherche, Rue de la Chine, Paris, 75020 France; 2 UCL Elizabeth Garrett Anderson Institute for Women’s Health, 74 Huntley Street, London, WC1E 6AU UK; 3 SAMU 93 - SMUR Pédiatrique, CHI André Gregoire, Groupe Hospitalier Universitaire Paris Seine-Saint-Denis, Assistance Publique des Hôpitaux de Paris, Montreuil, France; 4 Maternité Olympe de Gouges, Centre Hospitalier Regional Universitaire Tours, Tours, France; 5 Université François Rabelais, Tours, France; 6 UPMC Université Paris 6, Sorbonne Universités, Paris, France; 7 Service de Néonatologie, Hopital Armand Trousseau, Assistance Publique des Hôpitaux de Paris, Paris, France; 8 Maternité Port-Royal, University Paris-Descartes, Hôpitaux Universitaires Paris Centre, Assistance Publique des Hôpitaux de Paris, Paris, France; 9 URC CIC P1419, DHU Risk in Pregnancy, Cochin Hotel Dieu, Assistance Publique des Hôpitaux de Paris, Paris, France

**Keywords:** Extreme prematurity, Newborn, Perinatal intensity, Activity, Obstetric, Neonatal, Epidemiology, Cohort study, Health services organisation, Neonate

## Abstract

**Background:**

Perinatal decision-making affects outcomes for extremely preterm babies (22–26 weeks’ gestational age (GA)): more active units have improved survival without increased morbidity. We hypothesised such units may gain skills and expertise meaning babies at higher gestational ages have better outcomes than if they were born elsewhere. We examined mortality and morbidity outcomes at age two for babies born at 27–28 weeks’ GA in relation to the intensity of perinatal care provided to extremely preterm babies.

**Methods:**

Fetuses from the 2011 French national prospective EPIPAGE-2 cohort, alive at maternal admission to a level 3 hospital and delivered at 27–28 weeks’ GA, were included. Morbidity-free survival (survival without sensorimotor (blindness, deafness or cerebral palsy) disability) and overall survival at age two were examined. Sensorimotor disability and Ages and Stages Questionnaire (ASQ) result below threshold among survivors were secondary outcomes. Perinatal care intensity level was based on birth hospital, grouped using the ratio of 24–25 weeks’ GA babies admitted to neonatal intensive care to fetuses of the same gestation alive at maternal admission. Sensitivity analyses used ratios based upon antenatal steroids, Caesarean section, and newborn resuscitation. Multiple imputation was used for missing data; hierarchical logistic regression accounted for births nested within centres.

**Results:**

633 of 747 fetuses (84.7%) born at 27–28 weeks’ GA survived to age two. There were no differences in survival or morbidity-free survival: respectively, fully adjusted odds ratios were 0.96 (95% CI: 0.54 to 1.71) and 1.09 (95% CI: 0.59 to 2.01) in medium and 1.12 (95% CI: 0.63 to 2.00) and 1.16 (95% CI: 0.62 to 2.16) in high compared to low-intensity hospitals. Among survivors, there were no differences in sensorimotor disability or ASQ below threshold. Sensitivity analyses were consistent with the main results.

**Conclusions:**

No difference was seen in survival or morbidity-free survival at two years of age among fetuses alive at maternal hospital admission born at 27–28 weeks’ GA, or in sensorimotor disability or presence of an ASQ below threshold among survivors. There is no evidence for an impact of intensity of perinatal care for extremely preterm babies on births at a higher gestational age.

## Background

Extremely preterm babies, defined as those born at a gestational age (GA) between 22 and 26 weeks, have benefited from the introduction of evidence-based management strategies leading to improved outcomes. These include the administration of antenatal steroids, appropriate early respiratory management, and prevention of neonatal hypothermia following delivery, as well as organisational changes to promote delivery in a unit with appropriate neonatal facilities [1].

Decision-making at these gestations remains an important determinant of both mortality and morbidity, with substantial international variability in the management of these (threatened) extremely preterm deliveries occurring both ante- and post-natally [2, 3]. In France, there is no disagreement that babies of 27 weeks’ GA or higher should be provided with active care [4, 5]. In contrast, there is substantial variability in the approach taken at different hospitals to the resuscitation of babies born at 24 or 25 weeks’ gestation [6, 7]. Using data from the French national cohort study, EPIPAGE-2, initiated in 2011 [8], we created an indicator that measured the intensity of active perinatal care at a hospital level and not just which treatments were administered to the mother or baby. With this indicator, we demonstrated that delivery at less than 27 weeks’ gestation in hospitals with a higher intensity of perinatal care is associated with improved survival without any difference in sensorimotor outcomes at two years of age [9]. Similar findings have been obtained using measures of perinatal activity based on specific obstetric and neonatal treatments at a regional level [10] and, using only neonatal indicators, at a hospital level [11].

It is less clear whether there is an effect of the intensity of perinatal care for extremely preterm babies on those who are born at higher gestational ages – for whom there is much greater consensus in terms of perinatal management. Specifically, units that are more active in their care for extremely preterm babies may develop expertise that also leads to improved outcomes for babies born at a higher gestational age. A study examining this question among live born babies demonstrated improved outcomes at hospital discharge [12]. The “perinatal interventional activity score”, however, was partly based upon obstetric measures and thus did not take into consideration treatments for fetuses who died during labour. Another study examined babies born at 25 to 27 weeks’ gestation and also found improved outcomes [13], but disproportionately included babies born small for gestational age meaning it is difficult to generalise the results.

In this study, we examine whether there are differences in survival and sensorimotor disability at two years of age for babies born at 27 and 28 weeks’ gestation in relation to the intensity of perinatal care provided to extremely preterm babies born in France in 2011. We hypothesised that there would be higher rates of survival without increases in morbidity for babies born at 27–28 weeks’ gestation in hospitals that had a higher intensity of perinatal care for babies born extremely preterm.

## Methods

### Study population

Case identification, data collection and other design aspects for the EPIPAGE-2 cohort have been described previously [8]. In brief, all births between 22 and 26 completed weeks of gestation (i.e. 26 weeks and 6 days or less) collected over an 8 month period and all those at 27–28 weeks’ gestation collected over 6 month period were included [8]. For this study, the baseline population comprised all births at 27 to 28 weeks’ gestation occurring in a level 3 hospital [14] with at least one delivery at 24 or 25 weeks’ gestation. We excluded fetuses that were not alive at maternal admission to hospital and at either the start of monitoring of the labour or when it was decided to perform Caesarean section, as well as those fetuses with congenital lethal malformations; terminations of pregnancy for congenital anomalies were also excluded. Data were included only if parental consent for inclusion was received.

### Outcomes

The primary outcome was morbidity-free survival at two years of age, defined as those surviving babies who were free from sensorimotor disability; we also examined overall survival. Secondary outcomes were sensorimotor disability at two years of age among survivors, a combined outcome consisting of cerebral palsy (motor) or sensory disability, and neurodevelopmental status.

Sensory disability (blindness in one or both eyes and/or unilateral or bilateral deafness) and cerebral palsy were assessed by the attending physician; cerebral palsy was defined according to the diagnostic criteria of the Surveillance of Cerebral Palsy in Europe (SCPE) network with independent review of ambiguous cases by a committee of experts [15]. Neurodevelopment was assessed using the second version of the Ages and Stages Questionnaire (ASQ) completed by parents; data were included if completed between 22 and 26 months corrected age in children without cerebral palsy or sensory disability (deafness and blindness), and who did not have a severe brain malformation. Data covered five developmental domains: communication, gross motor, fine motor, problem-solving and personal-social; an ASQ score below threshold was defined for those children who scored lower than two standard deviations from the mean in at least one domain [16].

### Intensity of active perinatal care

**Fig. 1:**
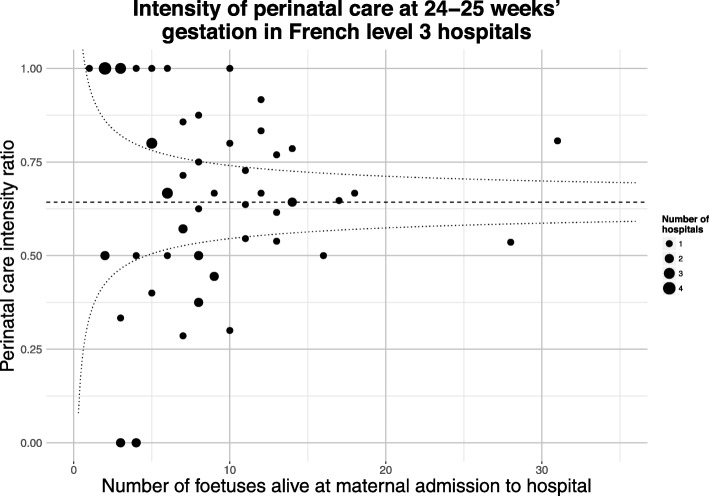
Intensity of perinatal care at 24–25 weeks’ gestation in French level 3 hospitals. Perinatal intensity is calculated as the ratio of babies born at 24–25 weeks’ gestational age who were admitted into neonatal intensive care divided by the number of fetuses delivered at the same gestational age who were alive at maternal admission to hospital or when the decision to perform Caesarean section was made; weighted average intensity is indicated with a dashed line, 25^th^ and 75^th^ percentile limits with dotted lines (Reproduced from Morgan et al, BMC Medicine (2018) 16:227 [9])

Care provided by teams at different hospitals was categorised into three groups using “perinatal intensity” ratios. These have been previously described [9] and were based on the number of babies of 24–25 weeks’ gestation admitted into neonatal intensive care divided by the number of fetuses alive at maternal admission to hospital and subsequently delivered at 24–25 weeks’ gestation. The average intensity, weighted according to the number of viable fetuses admitted to hospital, was used to identify 25^th^ and 75^th^ percentile limits [17]. This accounted for increased variability around estimates for hospitals with few admissions at 24–25 weeks’ gestation (thus addressing the concern that the intensity ratio for smaller hospitals may be imprecise). Using these limits, we created a “low” intensity group containing 19 hospitals, a “medium” intensity group containing 20 hospitals, and a “high” intensity group containing 23 hospitals, as shown in Fig. 1 reproduced from our previous manuscript [9].

### Potential explanatory variables

Data were available for maternal, pregnancy and neonatal factors. Maternal characteristics considered were: age (less than 25, 25–29, 30–34, 35 and over), parity (number of previous viable births), country of birth (France or another country), and socioeconomic status (defined according to the highest occupational status of both parents, or mother only if it was a single parent family, and divided into six categories: professional; intermediate; administrative, public service, self-employed, students; shop assistants, service workers; manual workers; unemployed). In relation to the current pregnancy, there was information on fertility treatment, singleton or multiple pregnancy, fetal sex, presence of clinically diagnosed chorioamnionitis, whether there was premature prolonged rupture of membranes (pPROM, defined as occurring more than 12 hours prior to delivery), if there was a spontaneous onset of labour, gestational age at delivery (in completed weeks’ gestation), and fetal presentation. For babies, birth weight z-score (using French “EPOPé” intrauterine growth curves[18]) was available.

### Statistical methods

We first described mortality and morbidity outcomes for babies born at 27–28 weeks’ gestation in the three groups of hospitals. We then identified crude associations of potential explanatory variables with perinatal intensity levels through cross-tabulation.

We carried out all subsequent analyses using imputed data due to missing data, particularly for the outcome variables collected at two years of age. As described previously, the imputed data sets were created using variables that potentially predicted non-response or the outcome [9, 15]. We used 27 variables in the imputation models, including both the exposures and the two year outcomes, as well as background maternal, pregnancy and neonatal variables; further details are provided in Additional file 1. For the main analyses, we performed analysis between the assigned intensity level and the outcome using multilevel logistic regression with clustering at the level of the hospital to provide an unadjusted estimate of the association. Similar to the strategy in our previous paper [9], we amended this model by sequentially adding gestational age at delivery (model 2), multiple pregnancy status (model 3), and then extra variables (model 4). These were variables considered a priori to be potential confounders: maternal age, family socio-economic status, fertility treatment during the current pregnancy, chorioamnionitis, pPROM, spontaneous labour, fetal sex, and fetal size at delivery. A p-value of <0.05 was considered as statistically significant for all analyses. All statistical analyses were conducted using R version 3.3.3, [19], with the package ‘mice’ [20] used for multiple imputation.

### Sensitivity analyses

As this hypothesis has previously been studied using hospital rates of antenatal steroid administration, Caesarean section and neonatal resuscitation, we constructed indicators based on the use of these factors for babies delivered at 24 to 25 weeks’ gestation, weighted in a similar fashion to our perinatal intensity indicator. A detailed description of the construction of these indicators is provided in Additional file 1. We then examined the impact of these indicators on sensorimotor disability and neurodevelopmental impairment among survivors. We also used our main indicator of perinatal intensity to look at these outcomes in cases with complete data to ensure results were coherent with our main analyses.

## Results

**Fig. 2:**
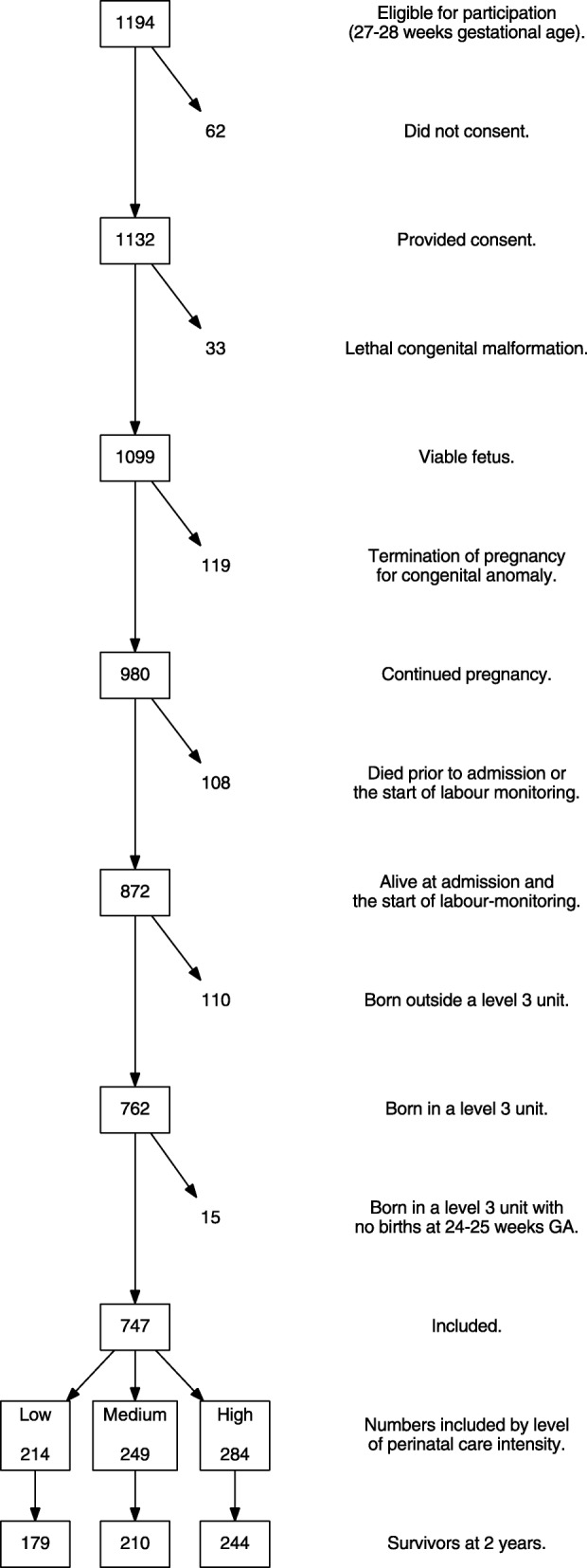
Study population. Flow chart of 27–28 weeks’ gestational age births from the EPIPAGE-2 cohort included in the study population at two years corrected age

Consent was provided for 1132 of 1194 births occurring at 27–28 weeks’ gestational age in France during the six month study period in 2011. Of these, 872 were alive at admission and at the onset of labour-monitoring (or when a decision was made to perform Caesarean section); 110 were born outside a level 3 with five of the six babies transferred postnatally and 78 of the 104 not transferred surviving to discharge. Overall, 747 babies met the inclusion criteria and were born in a level 3 hospital with at least one birth at 24 to 25 weeks’ gestation (Fig. 2). There were 214 births in hospitals classified as having low perinatal intensity, 249 in hospitals of medium intensity and 284 in high-intensity hospitals. No important differences were seen between groups in terms of population characteristics (Tables 1 and 2 in Additional file 2). Survival rates at two years corrected age were similar (83.6%, 84.3% and 85.9% in low, medium and high activity hospitals, respectively), as were rates of sensorimotor disability and ASQ scores below threshold in complete cases and imputed populations (Table 1). However, of the 633 survivors, only 539 (85.2%) had information available relating to sensorimotor deficiency and 402 (63.6%) for the Ages and Stages Questionnaire.

**Table 1:** Numbers and percentages with confidence intervals by level of intensity

	Perinatal intensity level								
	Low			Medium			High		
	n	%	(95%CI)	n	%	(95%CI)	n	%	(95%CI)
Fetal admissions	214	(— reference —)		249	(— reference —)		284	(— reference —)	
Live born	209	97.7	(94.3 – 99.1)	239	96.0	(92.5 – 97.9)	271	95.4	(92.1 – 97.4)
Admitted to NICU	208	97.2	(93.7 – 98.9)	236	94.8	(91 – 97.1)	268	94.4	(90.8 – 96.6)
Survived to 2 years	179	83.6	(77.8 – 88.2)	210	84.3	(79.1 – 88.5)	244	85.9	(81.2 – 89.6)
CP (n responding)	153	(— reference —)		175	(— reference —)		211	(— reference —)	
CP/sensory deficiency	5	3.3	(1.2 – 7.9)	14	8.0	(4.6 – 13.3)	13	6.2	(3.5 – 10.5)
ASQ (n responding)	113	(— reference —)		127	(— reference —)		162	(— reference —)	
ASQ < threshold	44	38.9	(30 – 48.6)	62	48.8	(39.9 – 57.8)	69	42.6	(34.9 – 50.6)
*Imputed* *population*^∗^	179	–	–	210	–	–	244	–	–
*CP*/*sensorydeficiency*^∗^	–	4.4	(3.0 – 5.8)	–	8.6	(6.8 – 10.4)	–	6.5	(5.1 – 8.0)
*ASQ*<*threshold*^∗^	–	47.5	(44.0 – 50.9)	–	54.1	(50.9 – 57.3)	–	47.1	(44.1 – 50.1)

^∗^Imputed percentages were averaged across the 60 imputed data sets using Rubin’s rule [21]

### Morbidity-free survival

There were no differences between groups in terms of survival or survival without sensorimotor morbidity, as shown in Table 2. Fully adjusted analyses showed ORs of 0.96 (95% CI: 0.54 to 1.71) and 1.09 (95% CI: 0.59 to 2.01) in medium intensity hospitals for survival and survival without sensorimotor morbidity, respectively, and the corresponding ORs in high intensity hospitals were 1.12 (95% CI: 0.63 to 2.00) and 1.16 (95% CI: 0.62 to 2.16).

**Table 2:** Odds ratios for outcomes at 2 years of age (cerebral palsy (CP) and sensory deficiencies (blindness and deafness), and Ages and Stages Questionnaire (ASQ) results below threshold) amongst survivors of babies born at 27–28 weeks’ gestation in medium and high intensity units compared to low intensity units in France in 2011 using the Perinatal Activity Indicator based on babies born at 24–25 weeks’ GA

Model	Medium intensity		High intensity	
	OR	(95% CI)	OR	(95% CI)
Survival (among fetuses alive at maternal admission to hospital)				
Baseline	1.05	(0.64 – 1.75)	1.20	(0.72 – 1.97)
Baseline + GA	1.01	(0.61 – 1.67)	1.19	(0.72 – 1.95)
Baseline + GA + multiple status	0.99	(0.60 – 1.64)	1.15	(0.70 – 1.89)
Baseline + extra variables	0.96	(0.54 – 1.71)	1.12	(0.63 – 2.00)
Survival without sensorimotor morbidity (among fetuses alive at maternal admission to hospital)				
Baseline	1.04	(0.60 – 1.81)	1.39	(0.80 – 2.44)
Baseline + GA	1.25	(0.70 – 2.25)	1.32	(0.73 – 2.38)
Baseline + GA + multiple status	1.20	(0.67 – 2.14)	1.27	(0.70 – 2.28)
Baseline + extra variables	1.09	(0.59 – 2.01)	1.16	(0.62 – 2.16)
CP and sensory disability (among survivors)				
Baseline	2.04	(0.73 – 5.75)	1.53	(0.54 – 4.37)
Baseline + GA	2.11	(0.73 – 6.11)	1.54	(0.52 – 4.50)
Baseline + GA + multiple status	2.10	(0.72 – 6.15)	1.53	(0.52 – 4.53)
Baseline + extra variables	2.02	(0.66 – 6.13)	1.68	(0.53 – 5.28)
ASQ below threshold (among survivors)				
Baseline	1.27	(0.72 – 2.24)	0.98	(0.57 – 1.68)
Baseline + GA	1.30	(0.74 – 2.24)	0.99	(0.58 – 1.69)
Baseline + GA + multiple status	1.28	(0.72 – 2.28)	0.97	(0.56 – 1.67)
Baseline + extra variables	1.35	(0.76 – 2.40)	1.01	(0.58 – 1.76)

95% CI: 95% confidence interval. GA: gestational age. Extra variables: GA + multiple status + fetal sex + maternal age + family socioeconomic status + fertility treatment + chorioamnionitis + labour type + social security + small for GA + premature rupture of membranes. All analyses used multiple imputation

### Secondary outcomes

Sensorimotor disability was present in 32 of the 539 surviving children for whom information was available. After imputation, rates increased from 3.3% to 4.4%, 8.0% to 8.6% and 6.2 to 6.5% in the low, medium and high intensity groups, respectively. The proportion of children with an ASQ result below threshold increased from 38.9% to 47.5% in children born in a low intensity hospital, 48.8% to 54.1% and 42.6% to 47.1% in those born in those born in a medium and high intensity hospitals following imputation (Table 1). For both outcomes, there were no differences between the intensity groups in either unadjusted or adjusted analyses, as shown in Table 2.

### Sensitivity analyses

No differences were seen in the sensitivity analyses between hospitals of differing intensity level for either sensorimotor disability or neurodevelopmental impairment using indicators of perinatal activity based on rates of antenatal steroid administration, delivery by Caesarean section or resuscitation in the delivery room. Results are shown in Table 3.

**Table 3:** Fully adjusted odds ratios for outcomes at 2 years of age (cerebral palsy (CP) and sensory deficiencies (blindness and deafness), and Ages and Stages Questionnaire (ASQ) results below threshold) amongst survivors of babies born at 27–28 weeks’ gestation in medium and high intensity units compared to low intensity units in France in 2011 using indicators constructed in relation to births at 24–25 weeks’ gestation from rates of antenatal steroid exposure, delivery by Caesarean section and neonatal resuscitation in the delivery room

Model	Medium intensity		High intensity	
	OR	(95% CI)	OR	(95% CI)
CP and sensory disability (among survivors)				
Indicator created from rates of:				
Antenatal steroid exposure	1.10	(0.35 – 3.42)	1.16	(0.44 – 3.01)
Delivery by Caesarean section	0.98	(0.40 – 2.40)	0.49	(0.14 – 1.71)
Neonatal resuscitation in the DR	1.76	(0.59 – 5.25)	1.46	(0.50 – 4.33)
ASQ below threshold (among survivors)				
Indicator created from rates of:				
Antenatal steroid exposure	0.89	(0.50 – 1.60)	0.92	(0.55 – 1.57)
Delivery by Caesarean section	0.83	(0.50 – 1.37)	0.88	(0.50 – 1.55)
Neonatal resuscitation in the DR	1.59	(0.88 – 2.89)	1.19	(0.68 – 2.06)

95% CI: 95% confidence interval. DR = Delivery room. All analyses adjusted for gestational age, multiple status, fetal sex, maternal age, family socioeconomic status, fertility treatment, chorioamnionitis, labour type, social security, small for gestational age and premature rupture of membranes. All analyses used multiple imputation

There were substantial missing data in the complete case analyses. The final model for sensorimotor disability showed an important effect in medium intensity hospitals (OR 5.81 with a 95% CI: 1.18 to 28.48), but not in high intensity hospitals (OR 3.98, 95% CI: 0.78 to 20.22), although in both cases the confidence intervals were extremely wide. There was greater consistency between results of the complete case analyses for neurodevelopmental impairment with the results following imputation. Results are detailed in Additional file 2, Table 3.

## Discussion

### Principal findings

In this national population-based cohort study, we found no evidence that an increased intensity of perinatal activity for extremely preterm births is associated with improvements in survival or morbidity outcomes for babies born at a higher gestational age. Specifically, using a previously validated indicator, along with three variants based on markers used in other studies, we found no differences in the rates of morbidity-free survival, overall survival, sensorimotor disability, or ASQ scores below threshold for babies born in hospitals of low, medium or high intensity.

### Strengths and limitations of this study

This is the first study to investigate whether an increased intensity of perinatal active care for extremely preterm births is related to improved outcomes at a higher gestational age in a complete geographically-based cohort using the population of fetuses who are alive at both maternal admission to hospital and the onset of labour or when the decision was made to perform Caesarean section. This is a key point to emphasise, as these are the pregnancies in which it is possible to actively intervene to achieve a good outcome for the fetus, and eliminates the bias that may be introduced by focusing solely on live births [2, 3, 22].

The utility of the perinatal intensity indicator we used, which takes into consideration factors other than just the administration of specific treatments, has been previously demonstrated in the population of babies born below 27 weeks’ gestation [9]. This strength is enhanced as we validated the results obtained in the present study with this indicator by using multiple other indicators based on those used by others [13]. In all scenarios, the results were consistent, with no statistical evidence of an effect. While this does not exclude the possibility of an effect [23], odds ratios varied in both size and direction as might be expected when there is no true effect. Only one statistically significant result was found – in the complete case analysis, for surviving children who were born in hospitals of medium intensity. However, by using a p-value of <0.05, simply by chance one result in twenty would be expected to be significant. Furthermore, there were substantial missing data in the complete case analyses, likely causing an important selection bias. We used multiple imputation, including both the exposures and outcomes, as well as a range of other variables, in the imputation models. These were specifically chosen to ensure that the “missing at random” assumption was met for all covariates with missing data used in the main analyses [20, 24]; however, it can be difficult to know with certainty if the missing subjects are more or less likely to be affected by the outcomes under consideration [25].

In contrast, a potential weakness is that there were few children who had sensorimotor disability at two years of age, thus the study may have been lacking statistical power to identify an effect. This problem is reflected in wide confidence intervals for this outcome in both the imputed and complete case analyses. This is mitigated in two ways. First, there is a strong consistency in these results with the lack of effect seen among the other outcomes – particularly when considering the analysis for the primary outcome of survival without sensorimotor morbidity among fetuses alive at both maternal admission to hospital and the onset of labour monitoring (or decision to perform Caesarean section) where the point estimates tended very strongly towards the null. Secondly, there was a lack of consistency in the odds ratios obtained in the sensitivity analyses using different indicators, with the most prominent conclusion being that any effect is due to random error.

### Study findings in context

Two previous studies that examined the same hypothesis as this paper demonstrated better outcomes for babies born at a higher gestational age [12, 13]. However, both studies suffered from selection bias. In a US study, the included population was defined both by gestational age and by an upper weight limit of 1000 grams [13]. This meant that a substantial proportion of babies in the study (born at 25–27 weeks’ gestational age) would have been excluded as 1000g is around the 90^th^ centile for birth weight for babies born at 26 weeks and the 50^th^ centile for babies born at 27 weeks [26]. No differences were seen in relation to two indicators: rates of Caesarean section or neonatal resuscitation; and improvements in relation to unit rates of antenatal steroid use were only seen for combinations of death with neonatal morbidities [13].

A second study, conducted in Switzerland, included only live born babies yet their indicator included measures of obstetric activity [12]. Data were collected over eight years, meaning there may have been changes in attitude within centres during the study period. They found important differences in survival and the odds of major neonatal morbidities, weaker evidence for an effect on mortality and neurodevelopmental impairment combined, and no evidence of a difference in neurodevelopmental impairment amongst survivors at two years of age [12]. There is a clear coherence in these results with our study: both support the idea that there is no relationship between the intensity of perinatal activity provided to extremely preterm babies and outcomes at two years of age for those born at a higher gestational age.

We believe our findings have broader implications. Centralisation of care for extremely preterm babies has been shown to improve outcomes for those babies [1, 27, 28], but concerns have been raised that this may impact the acquisition of specialised knowledge or skills like intubation that also benefit other babies [29]. Similar concerns have been expressed in other domains such as paediatric intensive care [30] or in relation to transfer strategies for patients with major trauma or head injury [31]. Other services such as those for stroke [32], myocardial infarction [33] and oesophageal cancer surgery [34] have also been centralised, and similar questions might be asked. Our study shows that a lower level of experience with a high risk population – specifically, extremely preterm babies born in level 3 hospitals that are less active in their provision of care which may thus impact skills and knowledge – is not associated with long term consequences for other babies.

Finally, we note that the results from our study are representative of practices elsewhere. There is broad agreement in developed countries that all deliveries above 26 weeks should receive active perinatal care, whereas in 2011 there was greater variation at 24 and 25 weeks in France and elsewhere. For example, the attitudes of Dutch health care professionals varies most towards births at 24 and 25 weeks’ gestation [35], and the largest differences in survival seen in five European regions were at 24 weeks’ gestation, with much greater consistency in outcomes above this gestational age [36].

### Conclusion

This study examined the effect of intensity of perinatal care for extremely preterm births on the outcome of babies born at a higher gestational age. Using a previously validated indicator based on births at 24–25 weeks’ GA, we found no difference between groups in overall survival or survival without sensorimotor morbidity when considering the population of fetuses alive at maternal admission to hospital. We also found no differences in sensorimotor disability or of children scoring below threshold on the Ages and Stages Questionnaire for survivors born at 27–28 weeks’ GA in hospitals of differing perinatal intensity. We conclude that there is currently no evidence for an impact of the intensity of perinatal intensive care for extremely preterm babies on births at a higher gestational age.

## Supplementary information

**Additional file 1** S1 appendix: Supplementary methods.

**Additional file 2** S2 appendix: Supplementary results relating to intensity of perinatal care for extremely preterm babies and outcomes at a higher gestational age.

**Additional file 3** S3 appendix: STROBE checklist.

## Data Availability

Data used in the current study are not publicly available as they contain confidential information, but are available from the Scientific Group of the EPIPAGE 2 study for researchers who meet the criteria for access to confidential data on reasonable request.

## Abbreviations

GA: Gestational age
