# Supplementary material for: Intensity of perinatal care for extremely preterm babies and outcomes at a higher gestational age: evidence from the EPIPAGE-2 cohort study
Source: BMC Pediatr. 2020 Jan 7;20:8. doi: 10.1186/s12887-019-1856-1 (PMC6945524; doi:10.1186/s12887-019-1856-1)
Supplement: Supplementary file 1 — Additional file 1 S1 appendix: Supplementary methods. [file 12887_2019_1856_MOESM1_ESM.pdf]

RESEARCH

S1 Appendix: supplementary methods.

Andrei S Morgan[1,2,3*], Babak Khoshnood[1], Caroline Diguisto[1,6,7], Laurence Foix L'Helias[1,4,5], Laetitia Marchand-Martin[1], Monique Kaminski[1], Jennifer Zeitlin[1], Gérard Bréart[1], François Goffinet[1,8] and Pierre-Yves Ancel[1,9]

*Correspondence: andrei.morgan@inserm.fr
[1]Université de Paris, Epidemiology and Statistics Research Center/CRESS, INSERM (U1153 – Obstetrical, Perinatal and Pediatric Epidemiology Research Team (EPOPé)), INRA, Hôpital Tenon, Bâtiment Recherche, Rue de la Chine, 75020 Paris, France
Full list of author information is available at the end of the article

Multiple imputation

We used multiple imputation due to incomplete responses at two years of age which lead to missing data for both the main outcomes as well as covariates. Multiple imputation was performed using chained equations with the R package 'mice'.[1] Imputation model variables included the each of the possible exposures (i.e. perinatal intensity group using the primary as well as sensitivity indicators), two year outcomes (survival status, sensorimotor disability and neurodevelopmental impairment), maternal variables (age, parity, country of birth, socioeconomic status), pregnancy variables (fertility treatment, multiple status, fetal sex, chorioamnionitis, prolonged rupture of membranes, spontaneous labour, gestational age at delivery), birth weight z-score, neonatal morbidities (bronchopulmonary dysplasia, necrotising enterocolitis and retinopathy of prematurity) as well as usage of postnatal steroids, whether the baby received maternal breast milk at discharge, and whether there was a severe congenital brain malformation. Neurodevelopmental impairment data corresponded to the five domains (communication, gross motor, fine motor, problem-solving and personal-social) of the Ages and Stages Questionnaire (ASQ)[2] if completed between 22 and 26 months corrected age in children without cerebral palsy or neurosensory disability (deafness and blindness), and who did not have a severe brain malformation. Data for sensorimotor disability and ASQ status were only imputed for children who survived to two years of age. We generated 60 independent data sets using 30 iterations each; data were pooled according to Rubin's rule.[3]

Table 1: Multiple imputation variables: methods and missingness

Variable	Variable type	Model for data prediction	Percentage of values missing	
			foetuses	survivors
Hospital of birth	Categorical	No missing data	0%	0%
Intensity of perinatal care group *	Categorical (3 categories)	No missing data	0%	0%
Survival status	Categorical (6 categories)	No missing data	0%	0%
Gestational age (weeks)	Numeric	No missing data	0%	0%
Multiple pregnancy status	Binary	No missing data	0%	0%
Fetal sex	Binary	No missing data	0%	0%
Maternal age	Categorical (4 categories)	Ordered logit model	0.1%	0%
Parents' socioeconomic status	Categorical (6 categories)	Multinomial logit model	7.1%	5.8%
Maternal fertility treatment	Binary	Logistic regression	3.7%	3.5%
Chorioamnionitis	Binary	Logistic regression	9.1%	7.3%
Spontaneous labour	Binary	Logistic regression	6.7%	4.1%
Birth weight z-score	Numeric	Predictive mean matching	0.7%	0%
Premature rupture of membranes	Binary	Logistic regression	2.9%	1.6%
Maternal origin	Binary	Logistic regression	2.3%	1.1%
Primiparous	Binary	Logistic regression	0.8%	0.9%
Postnatal steroids	Binary	Logistic regression	8.4%	0.9%
Severe bronchopulmonary dypslasia	Binary	Logistic regression	13.8%	5.5%
Severe necrotising enterocolitis	Binary	Logistic regression	7.2%	2.1%
Severe retinopathy of prematurity	Binary	Logistic regression	5.8%	0.8%
Breastmilk at discharge	Binary	Logistic regression	22.0%	8.5%
Severe cerebral lesions	Binary	Logistic regression	7.0%	0.9%
Sensorimotor deficiency at 2 years CA	Binary	Logistic regression	27.8%	14.8%
ASQ Communication score below threshold at 2 years CA	Binary	Logistic regression	45.1%	35.2%
ASQ gross motor score below threshold at 2 years CA	Binary	Logistic regression	44.8%	34.9%
ASQ fine motor score below threshold at 2 years CA	Binary	Logistic regression	45.0%	35.1%
ASQ problem-solving score below threshold at 2 years CA	Binary	Logistic regression	45.6%	35.9%
ASQ personal-social score below threshold at 2 years CA	Binary	Logistic regression	45.2%	35.4%

* All four intensity indicators used in the main and sensitivity analyses were used for imputation.

Construction of the intensity indicators used in sensitivity analyses

We created three sensitivity indicators using a method similar to that we employed for the main indicator of perinatal intensity.[4] For each, hospitals were categorised into three levels, low, medium and high, according to a weighted calculation[5]based on the baseline population at 24 to 25 weeks' gestation. The three indicators were based on, respectively, the following treatments: fetal exposure to (any) antenatal steroids, delivery by Caesarean section, and active resuscitation in the delivery room (identified through the EPIPAGE-2 questionnaire completed at birth).

For each indicator, each level 3 hospital was assigned an activity ratio according to the number of fetuses or babies born 24 to 25 weeks' gestation who were exposed to the treatment divided by the number of fetuses alive at maternal admission to hospital who were subsequent born at 24 to 25 weeks' gestation (equation 1).

$$\text{Activity ratio } (p_i) = \frac{\text{Number of babies admitted into NICU}}{\text{Number of foetuses alive at maternal admission to hospital}} \quad (1)$$

Using these ratios, the mean activity level across all included hospitals was obtained using the formula shown in equation 2, where P_w is the overall weighted mean, p_i is the activity ratio in hospital i, and w_i is the weighting factor for hospital i:

$$P_w = \frac{\sum p_i w_i}{\sum w_i} \quad (2)$$

The weighting factors for individual hospitals were obtained using the formula shown in equation 3.

$$w_i = \frac{1}{\hat{\sigma}_p^2 + \frac{(\bar{p}(1-\bar{p})-\hat{\sigma}_p^2)}{n_i}} \quad (3)$$

In this equation, $\bar{p}$ represents the unweighted mean activity ratio of all hospitals (obtained simply by summation of all the ratios and dividing by the total number of hospitals), and $\hat{\sigma}_p^2$ is the estimated standard deviation, which is obtained from the following equation:

$$\hat{\sigma}_p^2 = \frac{\sum (p_i - \bar{p})^2}{k-1} - \frac{\sum \frac{p_i(1-p_i)}{n_i}}{k} \quad (4)$$

Here, again, $\bar{p}$ is the unweighted mean activity ratio, p_i is the activity ratio for hospital i, n_i is the number of fetuses alive at maternal admission to hospital in hospital i, and k is the total number of hospitals.

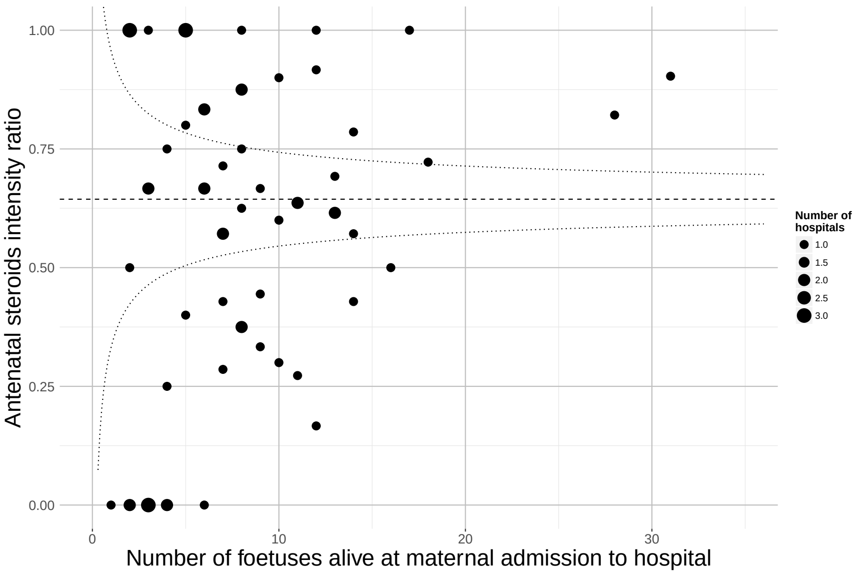

Figure 1: Intensity of perinatal care at 24–25 weeks gestation in French level 3 hospitals using an indicator constructed from fetal exposure to antenatal steroids.

Having calculated the mean activity level, 25th and 75th percentiles were obtained for different numbers of fetuses alive at maternal admission to hospital using equation 5:

$$\text{25th/75th percentiles} = P_w \pm 0.675\left(\frac{\sqrt{\hat{\sigma}_p^2}}{\sqrt{n}}\right) \tag{5}$$

where n is the number of foetuses admitted into hospital and $\hat{\sigma}_p^2$ is defined by equation 4. This enabled individual hospitals to be compared to the percentiles, and consequently permitting allocation to one of the three potential groups created. This is shown in figures 1, 2, and 3.

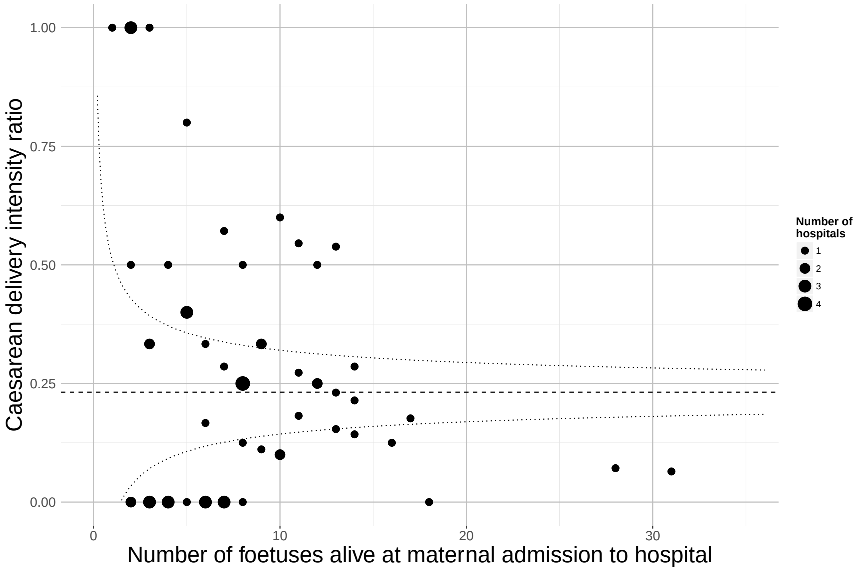

Figure 2: Intensity of perinatal care at 24–25 weeks gestation in French level 3 hospitals using an indicator constructed from the number of babies delivered by Caesarean section.

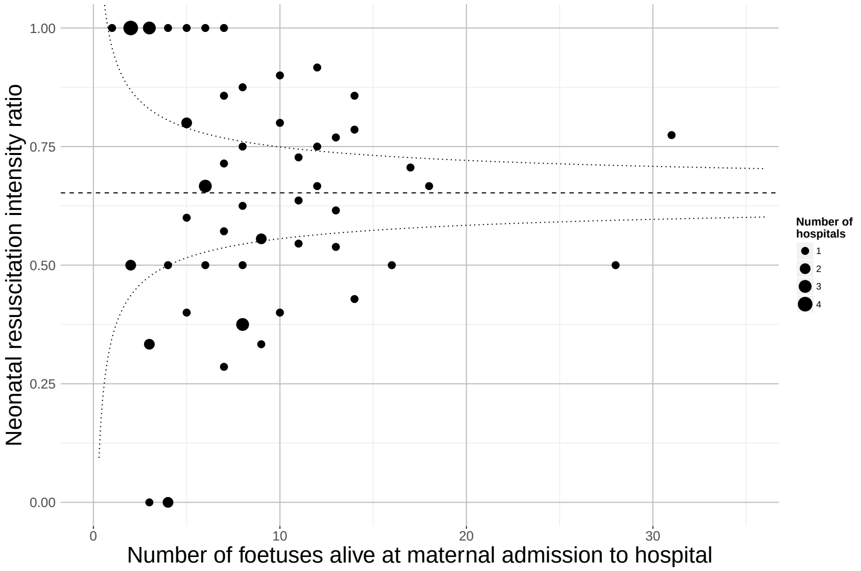

Figure 3: Intensity of perinatal care at 24–25 weeks gestation in French level 3 hospitals using an indicator constructed from the number of babies receiving active resuscitation at birth.

Author details
[1]Université de Paris, Epidemiology and Statistics Research Center/CRESS, INSERM (U1153 – Obstetrical, Perinatal and Pediatric Epidemiology Research Team (EPOPé)), INRA, Hôpital Tenon, Bâtiment Recherche, Rue de la Chine, 75020 Paris, France. [2]Institute for Womens' Health, UCL, 74 Huntley Street, WC1E 6AU, London, UK. [3]SAMU 93 - SMUR Pédiatrique, CHI André Gregoire, Groupe Hospitalier Universitaire Paris Seine-Saint-Denis, Assistance Publique des Hôpitaux de Paris, Montreuil, France. [4]UPMC Université Paris 6, Sorbonne Universités, Paris, France. [5] Service de Néonatologie, Hopital Armand Trousseau, Assistance Publique des Hôpitaux de Paris, Paris, France. [6] Maternité Olympe de Gouges, Centre Hospitalier Regional Universitaire Tours, Tours, France. [7] Université François Rabelais, Tours, France. [8] Maternité Port-Royal, University Paris-Descartes, Hôpitaux Universitaires Paris Centre, Assistance Publique des Hôpitaux de Paris, Paris, France. [9] URC CIC P1419, DHU Risk in Pregnancy, Cochin Hotel Dieu, Assistance Publique des Hôpitaux de Paris, Paris, France.

References

1. van Buuren, S., Groothuis-Oudshoorn, K.: mice: Multivariate Imputation by Chained Equations in R. Journal of Statistical Software, Articles **45**(3), 1–67 (2011). doi:10.18637/jss.v045.i03
2. Squires, J., Bricker, D., Twombly, E., Nickel, R., Clifford, J., Murphy, K., Hoselton, R., Potter, L., Mounts, L., Farrell, J.: Ages & Stages Questionnaires, Third Edition (ASQ-3). Brookes Publishing, ??? (2009)
3. Rubin, D.B.: Multiple Imputation for Nonresponse in Surveys. John Wiley & Sons, Inc., USA (1987). doi:10.1002/9780470316696.fmatter
4. Morgan, A.S., Foix L'Helias, L., Diguisto, C., Marchand-Martin, L., Kaminski, M., Khoshnood, B., Zeitlin, J., Breart, G., Durrmeyer, X., Goffinet, F., Ancel, P.-Y.: Intensity of perinatal care, extreme prematurity and sensorimotor outcome at 2 years corrected age: evidence from the epipage-2 cohort study. BMC Medicine **16**(1), 227 (2018). doi:10.1186/s12916-018-1206-4
5. Laird, N.M., Mosteller, F.: Some statistical methods for combining experimental results. International Journal of Technology Assessment in Health Care **6**(1), 5–30 (1990)
