# Supplementary material for: Intensity of perinatal care for extremely preterm babies and outcomes at a higher gestational age: evidence from the EPIPAGE-2 cohort study
Source: BMC Pediatr. 2020 Jan 7;20:8. doi: 10.1186/s12887-019-1856-1 (PMC6945524; doi:10.1186/s12887-019-1856-1)
Supplement: Supplementary file 2 — Additional file 2 S2 appendix: Supplementary results relating to intensity of perinatal care for extremely preterm babies and outcomes at a higher gestational age. [file 12887_2019_1856_MOESM2_ESM.pdf]

RESEARCH

S2 Appendix: supplementary results relating to intensity of perinatal care for extremely preterm babies and outcomes at a higher gestational age.

Andrei S Morgan[1,2,3*], Babak Khoshnood[1], Caroline Diguisto[1,6,7], Laurence Foix L'Helias[1,4,5], Laetitia Marchand-Martin[1], Monique Kaminski[1], Jennifer Zeitlin[1], Gérard Bréart[1], François Goffinet[1,8] and Pierre-Yves Ancel[1,9]

*Correspondence: andrei.morgan@inserm.fr
[1]Université de Paris, Epidemiology and Statistics Research Center/CRESS, INSERM (U1153 – Obstetrical, Perinatal and Pediatric Epidemiology Research Team (EPOPé)), INRA, Hôpital Tenon, Bâtiment Recherche, Rue de la Chine, 75020 Paris, France
Full list of author information is available at the end of the article

The following tables present data relating to the population of babies born at 27-28 weeks gestation according to the intensity of perinatal care for extremely preterm babies (born at 22-26 weeks' gestational age) in level 3 units in France in 2011 using data from the EPIPAGE 2 study.[1]

Author details
[1]Université de Paris, Epidemiology and Statistics Research Center/CRESS, INSERM (U1153 – Obstetrical, Perinatal and Pediatric Epidemiology Research Team (EPOPé)), INRA, Hôpital Tenon, Bâtiment Recherche, Rue de la Chine, 75020 Paris, France. [2]Institute for Womens' Health, UCL, 74 Huntley Street, WC1E 6AU, London, UK. [3]SAMU 93 - SMUR Pédiatrique, CHI André Gregoire, Groupe Hospitalier Universitaire Paris Seine-Saint-Denis, Assistance Publique des Hôpitaux de Paris, Montreuil, France. [4]UPMC Université Paris 6, Sorbonne Universités, Paris, France. [5] Service de Néonatologie, Hopital Armand Trousseau, Assistance Publique des Hôpitaux de Paris, Paris, France. [6] Maternité Olympe de Gouges, Centre Hospitalier Regional Universitaire Tours, Tours, France. [7] Université François Rabelais, Tours, France. [8] Maternité Port-Royal, University Paris-Descartes, Hôpitaux Universitaires Paris Centre, Assistance Publique des Hôpitaux de Paris, Paris, France. [9] URC CIC P1419, DHU Risk in Pregnancy, Cochin Hotel Dieu, Assistance Publique des Hôpitaux de Paris, Paris, France.

References

1. Ancel, P.-Y., Goffinet, F., Kuhn, P., Langer, B., Matis, J., Hernandorena, X., Chabanier, P., Joly-Pedespan, L., Lecomte, B., Vendittelli, F., Dreyfus, M., Guillois, B., Burguet, A., Sagot, P., Sizun, J., Beuchee, A., Rouget, F., Favreau, A., Saliba, E., Bednarek, N., Morville, P., Thiriez, G., Marpeau, L., Marret, S., Kayem, G., Durrmeyer, X., Granier, M., Baud, O., Jarreau, P.-H., Mitanchez, D., Boileau, P., Boulot, P., Cambonie, G., Daude, H., Bedu, A., Mons, F., Fresson, J., Vieux, R., Alberge, C., Arnaud, C., Vayssiere, C., Truffert, P., Pierrat, V., Subtil, D., D'Ercole, C., Gire, C., Simeoni, U., Bongain, A., Sentilhes, L., Roze, J.-C., Gondry, J., Leke, A., Deiber, M., Claris, O., Picaud, J.-C., Ego, A., Debillon, T., Poulichet, A., Coline, E., Favre, A., Flechelles, O., Samperiz, S., Ramful, D., Branger, B., Benhammou, V., Foix-L'Helias, L., Marchand-Martin, L., Kaminski, M.: Survival and morbidity of preterm children born at 22 through 34 weeks' gestation in France in 2011: results of the EPIPAGE-2 cohort study. JAMA Pediatrics **169**(3), 230–238 (2015). doi:10.1001/jamapediatrics.2014.3351

Table 1: Background factors relating to birth at 27-28 weeks' gestation in France in 2011 by exposure (intensity level of perinatal care for extremely preterm babies) group.

Variable	Levels	Low		Medium		High		P value
		n	%	n	%	n	%	
Gestational age (N=747)								
	27	105	49.1	106	42.6	136	47.9	0.312
	28	109	50.9	143	57.4	148	52.1	
Multiple pregnancy (N=747)								
	no	152	71.0	163	65.5	183	64.4	0.268
	yes	62	29.0	86	34.5	101	35.6	
Fetal sex (N=747)								
	male	96	44.9	126	50.6	138	48.6	0.461
	female	118	55.1	123	49.4	146	51.4	
Maternal age group (N=746)								
	<25 years	48	22.4	54	21.7	42	14.8	0.048
	25-29 years	59	27.6	73	29.3	97	34.3	
	30-34 years	49	22.9	67	26.9	88	31.1	
	>=35 years	58	27.1	55	22.1	56	19.8	
Mother born outside of France (N=730)								
	No	157	76.6	190	77.6	218	77.9	0.944
	Yes	48	23.4	55	22.4	62	22.1	
Single mother (N=687)								
	Yes	28	13.9	17	7.5	21	8.2	0.049
	No	174	86.1	211	92.5	236	91.8	
Maternal social security coverage (N=662)								
	No	17	8.9	34	15.6	32	12.6	0.129
	Yes	173	91.1	184	84.4	222	87.4	
Family SES (N=694)								
	Professional	39	19.8	35	15.2	52	19.6	0.009
	Intermediate	36	18.3	42	18.2	67	25.2	
	Administrative, public service, self-employed, students	55	27.9	67	29.0	70	26.3	
	Shop assistants, service workers	27	13.7	37	16.0	42	15.8	
	Manual workers	24	12.2	44	19.0	23	8.6	
	Unemployed	16	8.1	6	2.6	12	4.5	
Primiparous (N=741)								
	No	100	46.9	114	46.5	130	45.9	0.974
	Yes	113	53.1	131	53.5	153	54.1	
Maternal fertility treatment (N=719)								
	No	186	89.0	208	86.3	215	79.9	0.017
	Yes	23	11.0	33	13.7	54	20.1	
pPROM (N=725)								
	No	134	62.9	152	63.3	189	69.5	0.218
	Yes	79	37.1	88	36.7	83	30.5	
Chorioamnionitis diagnosed <48hrs to delivery (N=679)								
	No	159	79.9	167	77.0	216	82.1	0.373
	Yes	40	20.1	50	23.0	47	17.9	
Small for gestational age (N=742)								
	Yes	74	34.9	96	38.6	115	40.9	0.396
	No	138	65.1	153	61.4	166	59.1	
Spontaneous labour (N=697)								
	none	103	50.2	112	48.3	152	58.5	0.056
	spontaneous	102	49.8	120	51.7	108	41.5	
Spontaneous rupture of membranes (N=678)								
	No	100	50.0	106	46.1	139	56.1	0.090
	Yes	100	50.0	124	53.9	109	43.9	
Foetal presentation (N=712)								
	cephalic	122	59.5	145	61.7	155	57.0	0.645
	breech	71	34.6	78	33.2	95	34.9	
	other	12	5.9	12	5.1	22	8.1	

Table 2: Perinatal management factors relating to birth at 27-28 weeks' gestation in France in 2011 by exposure (intensity level of perinatal care for extremely preterm babies) group.

Variable	Levels	Low		Medium		High		P value
		n	%	n	%	n	%	
Antenatal steroids (N=724)								
	No	27	12.98	23	9.5	38	13.87	0.290
	Yes	181	87.02	219	90.5	236	86.13	
In utero transfer (N=730)								
	No	92	43.6	101	41.74	132	47.65	0.380
	Yes	119	56.4	141	58.26	145	52.35	
Tocolysis (N=729)								
	No	99	46.7	99	41.08	127	46.01	0.404
	Yes	113	53.3	142	58.92	149	53.99	
Maternal antibiotics (N=731)								
	No	101	47.64	114	46.91	159	57.61	0.025
	Yes	111	52.36	129	53.09	117	42.39	
MgSO4 usage (N=729)								
	No	191	89.67	214	89.17	258	93.48	0.174
	Yes	22	10.33	26	10.83	18	6.52	
Mode of delivery (N=722)								
	Caesarean	144	68.9	177	74.37	207	75.27	0.255
	Vaginal	65	31.1	61	25.63	68	24.73	
Baby resuscitated (N=701)								
	No	17	8.37	26	11.21	31	11.65	0.480
	Yes	186	91.63	206	88.79	235	88.35	

Table 3: Outcomes at two years of age (cerebral palsy (CP) and sensory deficiencies (blindness and deafness), and Ages and Stages Questionnaire (ASQ) results below threshold) amongst survivors of babies born at 27-28 weeks' gestation in medium and high intensity units compared to low intensity units in France in 2011 using perinatal activity indicator based on babies born at 24-25 weeks' gestation (subjects with complete data only).

Model	n	Medium intensity		High intensity	
		OR	(95% CI)	OR	(95% CI)
**CP/sensory deficiency**					
Unadjusted	539	2.40	(0.75 – 7.72)	1.86	(0.58 – 5.99)
Baseline + GA	539	2.54	(0.79 – 8.15)	1.90	(0.60 – 6.06)
Baseline + GA + multiple status	539	2.52	(0.79 – 8.11)	1.87	(0.59 – 6.00)
Baseline + extra variables	411	5.81	(1.18 – 28.48)	3.98	(0.78 – 20.22)
**ASQ below threshold**					
Unadjusted	402	1.54	(0.85 – 2.80)	1.14	(0.65 – 2.01)
Baseline + GA	402	1.63	(0.88 – 2.99)	1.14	(0.64 – 2.03)
Baseline + GA + multiple status	402	1.55	(0.83 – 2.89)	1.05	(0.58 – 1.91)
Baseline + extra variables	316	1.46	(0.73 – 2.94)	0.83	(0.42 – 1.64)

95% CI: 95% confidence interval. GA: gestational age. Extra variables: GA + multiple status + fetal sex + maternal age + family socioeconomic status + fertility treatment + chorioamnionitis + labour type + small for GA + premature rupture of membranes.
