# Supplementary material for: Intensity of perinatal care for extremely preterm babies and outcomes at a higher gestational age: evidence from the EPIPAGE-2 cohort study
Source: BMC Pediatr. 2020 Jan 7;20:8. doi: 10.1186/s12887-019-1856-1 (PMC6945524; doi:10.1186/s12887-019-1856-1)
Supplement: Supplementary file 3 — Additional file 3 S3 appendix: STROBE checklist. [file 12887_2019_1856_MOESM3_ESM.pdf]

RESEARCH

S3 Appendix: STROBE checklist

Andrei S Morgan[1,2,3*], Laurence Foix L'Helias[1,4,5], Caroline Diguisto[1,6,7], Laetitia Marchand-Martin[1], Monique Kaminski[1], Babak Khoshnood[1], Jennifer Zeitlin[1], Gérard Bréart[1], François Goffinet[1,8] and Pierre-Yves Ancel[1,9]

*Correspondence: andrei.morgan@inserm.fr
[1]Université de Paris, Epidemiology and Statistics Research Center/CRESS, INSERM (U1153 – Obstetrical, Perinatal and Pediatric Epidemiology Research Team (EPOPé)), INRA, Hôpital Tenon, Bâtiment Recherche, Rue de la Chine, 75020 Paris, France
Full list of author information is available at the end of the article

The following table contains the STROBE checklist of items to be included in reports of cohort studies[1] alongside a reference to where in the article the information may be found.

STROBE checklist for "Intensity of perinatal care for extremely preterm babies and outcomes at a higher gestational age: evidence from the EPIPAGE-2 cohort study

	Item No	Recommendation	Section (notes)
**Title and abstract**	1	(a) Indicate the study's design with a commonly used term in the title or the abstract	Title ("cohort study").
		(b) Provide in the abstract an informative and balanced summary of what was done and what was found	Abstract: Methods and Results sections.
**Introduction**			
Background/ rationale	2	Explain the scientific background and rationale for the investigation being reported	Background (paragraphs 1 to 3).
Objectives	3	State specific objectives, including any prespecified hypotheses	Background (final paragraph).
**Methods**			
Study design	4	Present key elements of study design early in the paper	Methods section.
Setting	5	Describe the setting, locations, and relevant dates, including periods of recruitment, exposure, follow-up, and data collection	Methods ("Study population" section).

Continued on next page...

Strobe checklist (continued)

	Item No	Recommendation	Section (notes)
Participants	6	(a) Give the eligibility criteria, and the sources and methods of selection of participants. Describe methods of follow-up	Methods ("Study population" and "Outcomes" sections).
		(b) For matched studies, give matching criteria and number of exposed and unexposed	N/A.
Variables	7	Clearly define all outcomes, exposures, predictors, potential confounders, and effect modifiers. Give diagnostic criteria, if applicable	Methods (sections on Outcomes, Intensity of perinatal care, Potential explanatory variables).
Data sources/ measurement	8[a]	For each variable of interest, give sources of data and details of methods of assessment (measurement). Describe comparability of assessment methods if there is more than one group.	Methods (sections on Outcomes, Intensity of perinatal care, Potential explanatory variables).
Bias	9	Describe any efforts to address potential sources of bias	Methods ("Statistical methods" section).
Study size	10	Explain how the study size was arrived at	Methods ("Study population") and Figure 2.
Quantitative variables	11	Explain how quantitative variables were handled in the analyses. If applicable, describe which groupings were chosen and why	Methods (Potential explanatory variables).
Statistical methods	12	(a) Describe all statistical methods, including those used to control for confounding	Methods (Statistical methods).
		(b) Describe any methods used to examine subgroups and interactions	Methods (Statistical methods, second paragraph).
		(c) Explain how missing data were addressed	Methods (Statistical methods, second paragraph) and S1 appendix: supplementary methods.
		(d) If applicable, explain how loss to follow-up was addressed	Methods (Statistical methods, second paragraph).
		(e) Describe any sensitivity analyses	Methods (Sensitivity analyses) and S1 appendix: supplementary methods.

Continued on next page...

Strobe checklist (continued)

	Item No	Recommendation	Section (notes)
**Results**			
Participants	13	(a) Report numbers of individuals at each stage of study — eg numbers potentially eligible, examined for eligibility, confirmed eligible, included in the study, completing follow-up, and analysed	Results (paragraph 1) and Figure 2.
		(b) Give reasons for non-participation at each stage	Figure 2.
		(c) Consider use of a flow diagram	Figure 2.
Descriptive data	14	(a) Give characteristics of study participants (eg demographic, clinical, social) and information on exposures and potential confounders	Results (paragraph 1), and S2 appendix: supplementary results relating to intensity of perinatal care for extremely preterm babies and outcomes at a higher gestational age (tables 1 and 2).
		(b) Indicate number of participants with missing data for each variable of interest	Results (first paragraph) and S2 appendix: supplementary results relating to intensity of perinatal care for extremely preterm babies and outcomes at a higher gestational age (tables 1 and 2).
		(c) Summarise follow-up time (eg, average and total amount)	N/A.
Outcome data	15	Report numbers of outcome events or summary measures over time	Results (first paragraph) and table 1.
Main results	16	(a) Give unadjusted estimates and, if applicable, confounder-adjusted estimates and their precision (eg, 95% confidence interval). Make clear which confounders were adjusted for and why they were included	Confounders are presented in the Methods (section "Statistical methods", paragraph 1). Unadjusted and adjusted estimates are presented in the Results as well as tables 2 and 3 and in S2 appendix: supplementary results relating to intensity of perinatal care for extremely preterm babies and outcomes at a higher gestational age (tables 1 to 3).
		(b) Report category boundaries when continuous variables were categorized	Methods ("Potential explanatory variables" section) and S2 appendix (tables 1 and 2).

Continued on next page...

Strobe checklist (continued)

	Item No	Recommendation	Section (notes)
		(c) If relevant, consider translating estimates of relative risk into absolute risk for a meaningful time period	N/A.
Other analyses	17	Report other analyses done — eg analyses of subgroups and interactions, and sensitivity analyses	Results (“Sensitivity analyses” section and S2 appendix: supplementary results relating to intensity of perinatal care for extremely preterm babies and outcomes at a higher gestational age, table 3).
**Discussion**			
Key results	18	Summarise key results with reference to study objectives	Discussion (paragraph 1)
Limitations	19	Discuss limitations of the study, taking into account sources of potential bias or imprecision. Discuss both direction and magnitude of any potential bias	Discussion (“Strengths and limitations” section).
Interpretation	20	Give a cautious overall interpretation of results considering objectives, limitations, multiplicity of analyses, results from similar studies, and other relevant evidence	Discussion (“Study findings in context” section) and conclusion.
Generalisability	21	Discuss the generalisability (external validity) of the study results	Discussion (“Study findings in context” section).
**Other information**			
Funding	22	Give the source of funding and the role of the funders for the present study and, if applicable, for the original study on which the present article is based	Funding section in the backmatter.

(a) Give information separately for exposed and unexposed groups.

Note: An Explanation and Elaboration article discusses each checklist item and gives methodological background and published examples of transparent reporting. The STROBE checklist is best used in conjunction with this article (freely available on the Web sites of PLoS Medicine at http://www.plosmedicine.org/, Annals of Internal Medicine at http://www.annals.org/, and Epidemiology at http://www.epidem.com/). Information on the STROBE Initiative is available at http://www.strobe-statement.org.

Author details
[1]Université de Paris, Epidemiology and Statistics Research Center/CRESS, INSERM (U1153 – Obstetrical, Perinatal and Pediatric Epidemiology Research Team (EPOPé)), INRA, Hôpital Tenon, Bâtiment Recherche, Rue de la Chine, 75020 Paris, France. [2]Institute for Womens' Health, UCL, 74 Huntley Street, WC1E 6AU, London, UK. [3]SAMU 93 - SMUR Pédiatrique, CHI André Gregoire, Groupe Hospitalier Universitaire Paris Seine-Saint-Denis, Assistance Publique des Hôpitaux de Paris, Montreuil, France. [4]UPMC Université Paris 6, Sorbonne Universités, Paris, France. [5] Service de Néonatologie, Hopital Armand Trousseau, Assistance Publique des Hôpitaux de Paris, Paris, France. [6] Maternité Olympe de Gouges, Centre Hospitalier Regional Universitaire Tours, Tours, France. [7] Université François Rabelais, Tours, France. [8] Maternité Port-Royal, University Paris-Descartes, Hôpitaux Universitaires Paris Centre, Assistance Publique des Hôpitaux de Paris, Paris, France. [9] URC CIC P1419, DHU Risk in Pregnancy, Cochin Hotel Dieu, Assistance Publique des Hôpitaux de Paris, Paris, France.

References

1. Vandenbroucke, J.P., von Elm, E., Altman, D.G., Gotzsche, P.C., Mulrow, C.D., Pocock, S.J., Poole, C., Schlesselman, J.J., Egger, M.: Strengthening the reporting of observational studies in epidemiology (STROBE): explanation and elaboration. PLoS Medicine **4**(10), 297 (2007). doi:10.1371/journal.pmed.0040297
